# Socio-Demographic and Lifestyle Factors Predict 5-Year Changes in Adiposity among a Group of Black South African Adults

**DOI:** 10.3390/ijerph14091089

**Published:** 2017-09-20

**Authors:** Cornelie Nienaber-Rousseau, Olusola F. Sotunde, Patricia O. Ukegbu, P. Hermanus Myburgh, Hattie H. Wright, Lize Havemann-Nel, Sarah J. Moss, Iolanthé M. Kruger, H. Salomé Kruger

**Affiliations:** 1 Centre of Excellence for Nutrition, North-West University, Potchefstroom 2520, South Africa; olusola.sotunde@mcgill.ca (O.F.S.); 30249864@nwu.ac.za (P.O.U.); 20266677@nwu.ac.za (P.H.M.); hwright@usc.edu.au (H.H.W.); lize.havemannnel@nwu.ac.za (L.H.-N.); salome.kruger@nwu.ac.za (H.S.K.); 2 School of Human Nutrition, McGill University, 21111 Lakeshore Road, Ste-Anne-de-Bellevue, Montréal, QC H9X 3V9, Canada; 3 Department of Human Nutrition and Dietetics, Michael Okpara University of Agriculture, Umudike PMB 7267, Abia State, Nigeria; 4 School of Health and Sports Sciences, University of the Sunshine Coast, Maroochydore, QSD 4558, Australia; 5 Physical Activity, Sport and Recreation Research Focus Area, North-West University, Potchefstroom 2520, South Africa; hanlie.moss@nwu.ac.za; 6 Africa Unit for Transdisciplinary Health Research, North-West University, Potchefstroom 2520, South Africa; lanthe.kruger@nwu.ac.za; 7 Medical Research Council Hypertension and Cardiovascular Disease Research Unit, North-West University, Potchefstroom Campus, Potchefstroom 2520, South Africa

**Keywords:** central obesity, marital status, marital transition, obesity, socio-demographic, socio-economic status, sub-Saharan Africa, urbanization

## Abstract

The rising prevalence of obesity and excessive adiposity are global public health concerns. Understanding determinants of changes in adiposity over time is critical for informing effective evidence-based prevention or treatment. However, limited information is available to achieve this objective. Cultural, demographic, environmental, and behavioral factors including socio-economic status (SES) likely account for obesity development. To this end, we related these variables to anthropometric measures in 1058 black adult Tswana-speaking South Africans who were HIV negative in a prospective study over five years. Body mass index (BMI) and waist circumference increased in both sexes, whereas triceps skinfold thickness remained the same. Over the five years, women moved to higher BMI categories and more were diagnosed with central obesity. Age correlated negatively, whereas SES, physical activity, energy, and fat intake correlated positively with adiposity markers in women. In men, SES, marital status, physical activity, and being urban predicted increases in adiposity. For women, SES and urbanicity increased, whereas menopause and smoking decreased adiposity. Among men, smokers had less change in BMI than those that never smoked over five years. Our findings suggest that interventions, focusing on the urban living, the married and those with the highest SES—the high-risk groups identified herein—are of primary importance to contain morbidity and premature mortality due to obesity in black South Africans.

## 1. Introduction

Obesity and excessive adiposity are global public health issues, and their prevalence seems to be ever increasing. This pandemic is associated with a myriad of co-morbidities—such as cardiovascular disease, type 2 diabetes mellitus as well as various cancers—and mortality [1]. The prevalence of obesity in South Africa is the highest in sub-Saharan Africa [2,3,4]. The 2012 South African Demographic and Health Survey (SADHS) reported an obesity (body mass index (BMI) > 30 kg/m^2^) prevalence of 11% and 41%, respectively, for both men and women over the age of 15, with the greatest contribution in the urban areas [4].

The etiology of obesity, and of excessive adiposity, is multifaceted and complex. Unraveling and understanding factors affecting this scourge are critical to halt and, ideally, reverse the problem. An appreciation of factors other than the biomedical—such as socio-cultural, demographic, environmental, and behavioral, including socio-economic status (SES), i.e., an individual’s position on a socio-economic scale measured through indicators such as education, income, occupation, and place of residence—are needed [2,5,6,7].

In developed countries, SES is inversely related to obesity [8,9], whereas in South Africa and other poor regions, the rich (those with the highest SES) are more likely to be overweight or obese than their less well-off counterparts elsewhere [4,6,7]. Having a low SES in low- and middle-income countries (LMIC) is associated with long-term weight gain, predicts BMI, and may contribute to an unfavorable body fat distribution [6,7]. Obesity in poor countries may be explained by overconsumption of energy-dense processed foods, which are relatively cheap and readily accessible [10,11,12]. Cultural aspects related to body image may also play a role. In many black African communities, being overweight or obese is regarded as a sign of good health, of beauty and affluence, whereas thinness is stigmatized due to its association with HIV/AIDS [2,13]. Moreover, lifestyle factors play a role in the degree of adiposity. For instance, sufficient physical activity is associated with a reduction in body fat among obese individuals, whereas increased levels of sedentarism are associated with obesity [14]. In addition, smoking has been associated with markers of non-communicable diseases (NCDs) including central fat accumulation [15], and in other studies with diminished measures of adiposity [7,16]. Quitting smoking seems to add to adiposity [17]. Previous studies reported conflicting results regarding the association of urbanicity versus rural residency on adiposity [18,19]. In high-income countries, the so-called rich nations, rural dwellers are reported to have higher adiposity, whereas the opposite is the case in LMICs, where urbanized individuals tend to be obese [18,19]. In South Africa, rapid urbanization is leading to the consumption of more westernized diets (comprising mainly energy-dense processed foods containing high amounts of fats [20]), eating meals away from home and insufficient physical activity levels [12,21] and, in turn, increases in obesity [4] that are mirrored by the greater prevalence of obesity-related NCDs [22].

Moreover, marital status circumscribes the social environment. In particular, being married seems to influence physical activity levels, food intake, and smoking habits [23,24,25,26]. Several studies point to its influence and highlighted the impact of transitions in and out of marriage on body weight [23,24,25,26]. However, information on marital status and transitions on markers of adiposity is lacking for black South Africans.

Numerous studies have been conducted on the associations of socio-demographic factors including measures of wealth on adiposity in Africa [5,6,16,27]. To our knowledge, none has focused on the predictors of adiposity, other than BMI and/or waist circumference (WC) over time, or investigated marital status or transitions among black South African adults, thus making the study reported here unique and original. The aim of this study is to fill a gap in the literature and extend current knowledge by exploring socio-demographic and SES characteristics at baseline in relation to adiposity over a period of five years. Furthermore, smoking and marital status were considered, and transitions in status over time were related to changes in adiposity measures over five years.

Adiposity can be assessed by anthropometry in several ways including by height, weight, BMI, waist and/or hip circumference and skinfold thickness measurements [28]. BMI, expressed as weight in relation to height, is often used as a proxy to evaluate levels of adiposity [29]. WC is a simple, yet sensitive, measure of central fat distribution and a good predictor of abdominal obesity [30]. Skinfold measurement is an inexpensive and accessible method of subcutaneous body fat assessment; in particular, the triceps site seems to be a valuable determinant of general obesity [31]. For our study, we used all the above measures to indicate adiposity. By not only determining BMI, we avoided the inherent limitation of BMI, which is that muscle mass can vary substantially between individuals of the same height and, therefore, has an imperfect association with body fat and is not always a true indicator of adiposity [32,33]. Additionally, the triceps skinfold is a marker of peripheral subcutaneous fat, whereas WC is abdominal, with both giving a more accurate reflection of total body adiposity, and both complement the BMI.

## 2. Materials and Methods

### 2.1. Study Design

**Figure 1:**
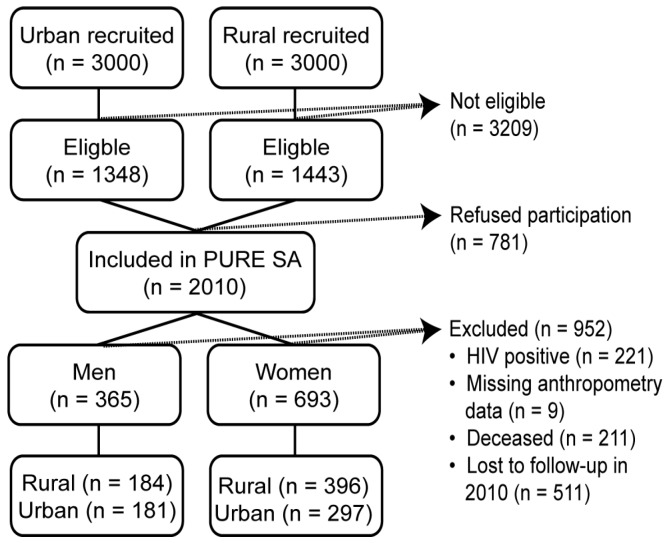
Consort diagram of the Prospective Urban and Rural Epidemiology (PURE) study reported herein.

This work is nested within the Prospective Urban and Rural Epidemiology (PURE) study, which is aimed at tracking the effects of lifestyle and changing environmental exposures on the development of NCDs [34]. The North West province arm of the PURE project in South Africa (PURE-SA) began with baseline data collected in 2005 and continued with follow-ups at five-year intervals. The current study reports on data collected during baseline and at the first follow-up. Owing to the large attrition rate, data from the third follow-up (in 2015) were not included. Recruitment procedures, study design, and methodology for PURE South Africa have been described in detail elsewhere [35]. Briefly, our sample included 2010 South African black adults older than 30 years and recruited from 6000 randomly selected households in two urban and two rural areas of the North West province. The urban communities were chosen from the established part of the township next to Potchefstroom, a major city in the North West province, and from the informal settlements that surround the township. The rural communities were identified in a remote area 450 km north-west of the city, in areas still under tribal law. Of the 2010 individuals participating at baseline, 722 were lost to follow-up, of whom 211 were deceased, as is shown in Figure 1.

We further excluded 221 HIV-positive participants and nine others with missing anthropometric data. Hence, a total sample of 1058 (365 men and 693 women) with complete data at baseline and at five-year follow-up were eligible for our study’s data analyses. Those who remained in their respective areas of residence throughout the study period were included, whereas change in address to outside the study areas (rural to urban or vice versa) was regarded as loss to follow-up. The participants lost to follow-up were younger and had significantly lower BMI, but a similar mean WC to those who were followed up [35]. We speculate that the participants lost to follow-up due to relocation could be a result of employment opportunities elsewhere for younger individuals with higher educational status. The study was approved by the Health Research Ethics Committee of North-West University (NWU), Potchefstroom campus (04M10 and NWU-00016-10-A1), South Africa. All participants provided written informed consent. The PURE-SA study conformed to the Declaration of Helsinki as revised in 2004.

### 2.2. Data Collection

#### 2.2.1. Questionnaires

Structured questionnaires were used by all countries participating in the PURE study to collect socio-demographic (including marital status) and lifestyle information at baseline [34]. The questionnaires also aided in the determination of menopausal status by asking women questions pertaining to the regularity of menses. Questionnaires were administered by trained fieldworkers during home visits and outings to the Metabolic Unit at NWU in their language of choice. Total fat and energy intakes were estimated from validated culturally sensitive quantitative food frequency questionnaires [36]. The nutrient intakes were coded and analyzed using the South African Medical Research Council’s food composition database [37]. A modified Baecke’s physical activity questionnaire, reported to be reliable and valid when compared with 24-h activity recalls among South Africa adults [38], was used. The physical activity questionnaire consisted of 21 questions organized into three sections: physical activity at work, organised sport, and activity during leisure time [36]. Three levels of occupational physical activity, namely low, middle, and high, were defined in the questionnaire. Questions in each of the indices were scored on a five-point Likert scale, ranging from “1 = never” to “5 = very often”. The sum of the four indices is the total physical activity score. A continuous physical activity score was calculated from the responses to the physical activity questionnaire. A score below 2.25 reflects the bottom tertile of physical activity and represents an occupation with no manual labor, commuting by public transport, no sports participation, and some light leisure-time activity, a score between 2.25 and 2.8 reflects a middle-level occupation, no organized sport, and some walking during leisure time, while the top tertile reflects a more active lifestyle with more leisure time activity and some sport participation [39].

#### 2.2.2. Socio-Economic Status Index

A uni-dimensional measure of SES was constructed by adapting a previously described SES index [40]. This index provides a better picture of the complex issue of SES and allowed us to observe poor-rich differences in adiposity measures. Our SES was calculated as the sum of the graded categories—for the educational level attained by the participants, type of occupation, source of household water, access to electricity and type of roofing material—at baseline. The SES index criteria were scored as follows: no formal education, 0; 1–7 years of formal education, 1; 8–12 years, 2; and more than 12 years, 3. For employment, being unemployed scored 0; domestic/informal work, 1; skilled work, 2; and professional work, 3. Source of water scored 1; if water was fetched from a river or dam, a community tap per street block, 2; and piped water inside the house, 3. No access to electricity scored 0, and access to electricity, 1. Use of informal roofing materials scored 1; asbestos, 2; galvanized iron, 3; and tiles, slates, or reinforced concrete, 4. The highest possible score was 14, and arbitrary scores between 2–4, 5–9, and 10–14 were allocated to indicate a low, moderate, and high SES, correlating to the tertiles of scores.

#### 2.2.3. Anthropometric Measurements

Anthropometric measurements were performed at baseline and follow-up according to standard methods of the International Society for the Advancement of Kinanthropometry. Height was measured to the nearest 0.1 cm with a stadiometer (Leicester height measure, Seca, Birmingham, UK) and weight was recorded on a portable electronic scale (Precision Health Scale, A & D Company, Kitamoto-shi, Saitama, Japan) to the nearest 0.01 kg with participants in light underwear and shoes removed. WC was measured at the narrowest point between the lower rib border and the iliac crest, and recorded to the nearest 0.1 cm with a steel tape (Lufkin, Cooper Tools, Apex, NC, USA). Abdominal obesity was defined by WC >94 cm for men and >80 cm for women [41]. Triceps skinfolds measurements were performed on the right arm of the participants with a Harpenden skinfold caliper (Baty International, West Sussex, UK), and the average of two recordings was used for data analysis. BMI was calculated by dividing weight in kilograms by height in meters squared and classified using the WHO categories of BMI, namely, <18.5 kg/m^2^ as underweight, 18.5–24.99 kg/m^2^ as normal weight, 25–29.99 kg/m^2^ as overweight and ≥30 kg/m^2^ as obese [1]. Relevant changes (∆) in adiposity variables were determined by subtracting adiposity values determined in 2005 from those recorded in 2010 for each individual, respectively.

### 2.3. Statistical Analysis

Normally distributed data are presented as means with standard deviation, and non-normally distributed data are presented as medians and interquartile range. Categorical data were analyzed using frequencies and prevalence of specific conditions and expressed as percentages.

Paired *t*-tests were used to assess the differences between baseline and follow-up values for energy and fat consumption as well as adiposity measures (BMI, WC, and triceps skinfold) for men and women according to residence (urban and rural). Because the literature indicates considerable differences in body composition between men and women [32], analyses were carried out separately for the sexes. We calculated the magnitude of changes in adiposity markers and presented these as effect sizes (Cohen’s *d*-values), i.e., the mean change over five years divided by the standard deviation of the baseline value [42]. Effect size calculation is a standard way of presenting magnitude of change over time. The BMI and abdominal obesity distributions of men and women in 2005 were compared with the corresponding values in 2010 using the Bhapkar test of equal category thresholds. This test was also used to compare the extent of changes in marital and smoking status between the two time points.

Spearman correlations were used to explore the relationship between baseline socio-economic variables, dietary intake and physical activity score, and changes in adiposity variables. Variables with significant correlations were entered in the full regression models (Model 1 for BMI change, WC change and triceps skinfold change for men and women, respectively—representing six separate regressions). Backwards multiple linear regressions were used to assess the association between baseline SES index, dietary intake and physical activity as predictors; and changes in three different adiposity variables over five years as the dependent variables, for men and women, separately. Potential confounders identified from the literature—namely, age, marital status, urbanization level, and menopausal status (for women only, cessation of menstrual periods via self-report)—were included in the models. In each case, Model 1 was the full model with all identified independent variables. Backwards regression was used in order to identify the relevant predictors that accounted for the most variance in the outcome variables, presented as the maximum R^2^ for the model. These identified determinants form Model 2, the best predicting model for each of the changes in adiposity markers.

Marital and smoking status transitions were also investigated, but were limited to those for whom we had marital and smoking status at the two time points and excluded those whose responses were missing (*n* = 65 for marital and *n* = 14 for tobacco use) or inconsistent from baseline to follow-up (*n* = 71) (as defined by Sobal et al. [24] for marital status and *n* = 87 for smoking status). Continuous marital status, transitions out of marriage, and transitions into marriage while never smoking, quitting smoking, starting smoking, and continuing smoking, respectively, were used as categories and other transitions were not included due to concerns about sample size. The associations of marital and smoking transition with change in adiposity markers were assessed with ANCOVA, stratified for sex while adjusting for age. Statistical significance was set at *p* < 0.05. Data were analyzed with IBM SPSS version 22 (IBM Company, Armonk, NY, USA), except for the Bhapkar test, which was performed using R software (https://cran.r-project.org/web/packages/irr/irr.pdf) (R Foundation for Statistical Computing, Vienna, Austria).

## 3. Results

### 3.1. Descriptive Baseline Data for the Sample Stratified According to Sex

Results of their 2005 baseline characteristics (Table 1) revealed that most adults (80.8%) reported primary school or no formal education, and 88.4% were domestic or informal workers. Women had significantly higher BMI, body weight, WC, and triceps skinfold values than men (*p* < 0.001). Men revealed a higher energy intake (*p* < 0.001), and more men than women were tobacco users (*p* < 0.001).

### 3.2. Five-Year Dietary and Adiposity Changes of Men and Women Stratified by Residence

#### 3.2.1. Changes in Dietary Intake

Dietary intakes differed between urban and rural residents for both sexes at baseline, with lower mean energy and fat intakes for rural dwellers than their urban counterparts (all *p* < 0.001). Over five years, energy and fat intakes increased in both rural and urban men and women. Changes in energy and fat intakes were higher for urban than for rural men (*p* = 0.003 and *p* = 0.04, respectively). Change in fat intake was less in urban than in rural women (*p* < 0.05), but the difference in energy intake change between urban and rural women was not significant (*p* > 0.05). Detailed analyses of dietary factors pertaining to adiposity were not within the scope of this investigation and were limited to factors known to change with urbanization.

#### 3.2.2. Changes in Adiposity

Over five years, men displayed a mean WC increase of 1 cm (*p* < 0.001) and a mean BMI increase of 0.42 kg/m^2^ (*p* < 0.001) with no change in mean triceps skinfold value (0.1 mm, *p* = 0.58). Similarly, mean WC of women enlarged by 2.3 cm (*p* < 0.001) and BMI by 0.90 kg/m^2^ (*p* < 0.001); however, mean triceps skinfold thickness remained unchanged (*p* = 0.72). The effect sizes—calculated as the mean change divided by standard deviation of the baseline values of the overall changes in BMI, WC, and triceps skinfold of both the men (*d* = 0.19, 0.09 and 0.16, respectively) and women (*d* = 0.2, 0.20 and 0.03, respectively)—were small (Table 2).

**Figure 2:**
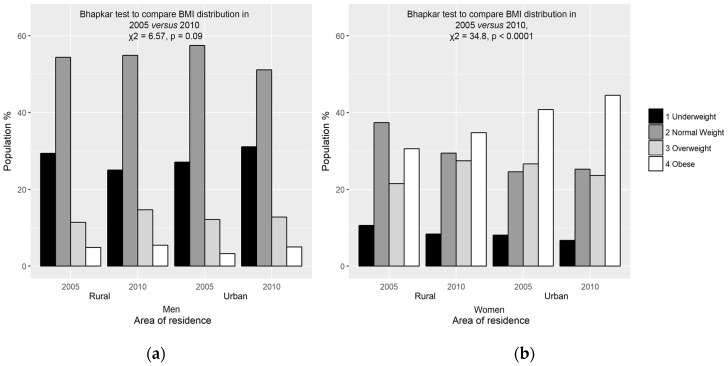
Adiposity status based on BMI of men (**a**) and women (**b**) stratified by residence at baseline and five-year follow-up.

The results of adiposity status based on BMI categories stratified by residence for men and women at the two time points are illustrated in Figure 2a,b. Greater agreement between baseline and follow-up categories was found in rural than in urban men (Figure 2a). Most rural (83.2%) and urban (78.9%) male subjects remained in the same BMI category over five years.

The Bhapkar tests for agreement between BMI subdivisions in 2005 and 2010 revealed that, although there was a trend to shift from underweight and normal weight to overweight and obese categories, the distribution among the categories at baseline and follow-up showed marginal homogeneity in the total group of men (χ^2^ = 6.57, *p* = 0.09). Less agreement was found in rural than among urban women. Almost three-quarters (74.6%) of rural and 79.5% of urban women were in the same BMI segment at both time points. Overall, there was a significant difference in distribution between the BMI categories of women in 2005 and 2010 (χ^2^ = 34.8, *p* < 0.0001), with a general shift from underweight and normal weight to overweight and obese categories (Figure 2b). Whereas most increases in BMI categories can be regarded as detrimental, moving from being underweight to normal weight is beneficial: a small group (5.48% men and 2.45% women) progressed from the underweight to normal weight BMI category.

**Figure 3:**
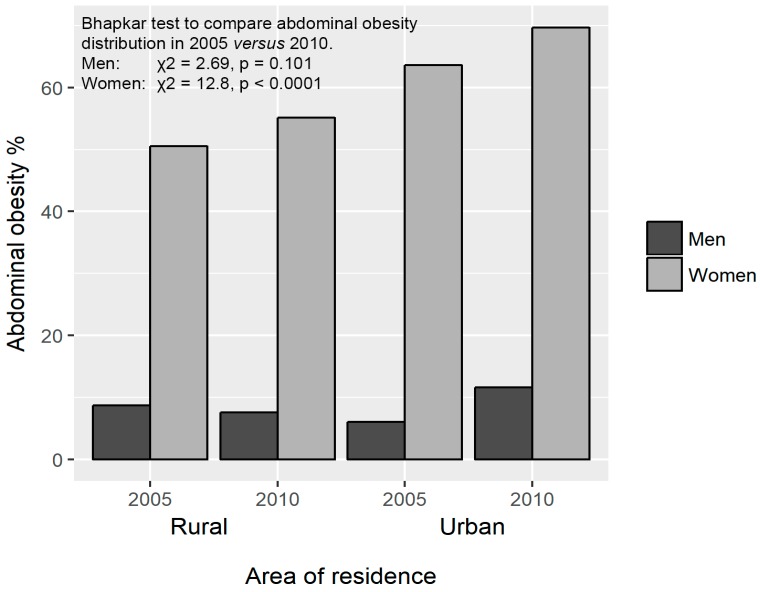
Abdominal obesity prevalence stratified by sex and residence at the two time points.

There was no difference in the prevalence of abdominal obesity in rural men at baseline and at follow-up (8.7% versus 7.6%). However, abdominal obesity increased in urban men from 6.1% to 11.7% over 5 years (*p* < 0.001). At baseline, 63.6% of urban women were identified with abdominal obesity compared to 50.5% of rural women (*p* = 0.001). At follow-up, abdominal obesity levels increased to 69.7% in urban and 55.1% in rural women (*p* < 0.001) (Figure 3). According to the Bhapkar test, the prevalence of abdominal obesity increased significantly over five years in women (χ^2^ = 12.8, *p* < 0.0001), but not in men (χ^2^ = 2.69, *p* = 0.10).

### 3.3. Association between Changes in Adiposity with Demographic, Socio-Economic and Lifestyle Variables

The Spearman correlation analysis revealed that none of the variables correlated with markers of adiposity in men (Table 3). Age was negatively correlated with changes in BMI (*r* = –0.16, *p* = 0.001) and with differences in triceps skinfold values (*r* = –0.12, *p* = 0.001), whereas the physical activity score at baseline was positively correlated with change in BMI (*r* = 0.13, *p* = 0.001) for women (Table 3). A positive correlation between change in WC and SES index (*r* = 0.14, *p* < 0.001) as well as level of education at baseline were established (*r* = 0.11, *p* = 0.001) for women (Table 3). Similarly, fat and energy intakes at baseline were also marginally positively correlated, albeit weakly, with changes in WC of women (*r* = 0.09, *p* < 0.05 for both).

### 3.4. Multivariate Analysis of Changes in Adiposity (Dependent) and Predictor (Baseline Socio-Economic and Lifestyle) Variables

Multiple linear regression analysis—for the association between changes in adiposity (dependent) and predictor (baseline demographic, socio-economic, and lifestyle factors, including dietary intake, physical activity, and tobacco use) variables—is presented in Table 4. In the final model (Model 2), using change in BMI as dependent variable, marriage (22%; *p* < 0.001) appeared to be the most attributable determinant followed by baseline physical activity score (12%; *p* < 0.05) among men. No baseline factors predicted changes in BMI among women.

The factors that determined changes in WC at baseline among men and women were baseline WC (*p* < 0.0001), SES (*p* = 0.03, both) and urban versus rural residence (*p* = 0.006 and *p* < 0.0001, respectively). Marital status predicted change in WC in men (accounting for 27% of the variance; *p* < 0.001), but not women. WC at baseline, SES index, marital status and area of residence explained 13.8% variation in WC change among men (Table 4; Model 2). A smaller percentage (10.7%) of variation in WC change of women was explained by baseline WC, age, SES and residential area (Table 4; Model 2).

Using triceps skinfold measurement as the dependent variable in the final model (Table 4; Model 2), SES, marital status and place of residence were positive predictors of change, whereas, baseline triceps skinfold thickness was a negative contributor for men. These determinants explained 23.1% of variation in triceps change. In women, baseline triceps values, menopausal status, and tobacco use were negative contributors, whereas SES was the only positive predictor of this adiposity marker, explaining 13.2% of triceps change (Table 4).

### 3.5. Marital Transitions and Changes in Adiposity

Even though the Bhapkar test indicated significant discrepancies between marital status in 2005 and 2010 (χ^2^ = 50.7; *p* < 0.0001), changes in markers of adiposity did not differ between constant marital status and alterations (transitioning in or out of marriage).

### 3.6. Smoking Status Changes and Changes in Adiposity

The Bhapkar tests revealed that smoking status differed from 2005 to 2010 for men (χ^2^ = 1008; *p* < 0.0001) and women (χ^2^ = 811; *p* < 0.0001), respectively. The relationship between the categories [i.e., never smoked (unchanged smoking status), stopped smoking (transitioning out of), started to smoke (transitioning into), continued to smoke (unchanged smoking status)] and the adiposity markers (change in BMI, WC, and triceps skinfold) were assessed while adjusting for age. For men, smoking status from 2005 to 2010 influenced change in BMI (*p* = 0.04). The *post hoc* Bonferroni test revealed that change in BMI for those that have never used tobacco differed significantly from continued users (*p* = 0.03). Continued smokers had less change in BMI [0.19 kg/m^2^; 95% CI (−0.097; 0.48)] over five years than those that never smoked [0.93 kg/m^2^; 95% CI (0.51; 1.35)].

## 4. Discussion

The novelty of our work lies in the identification of factors associated with increases in adiposity (not limited to BMI alone) over time and not just a mere snapshot of the determinants. In addition, we investigated for the first time marital status and the influences of transitions on markers of adiposity. Our study highlights the remarkable prevalence of excessive adiposity, especially among women, and showed an increase in BMI and WC with subcutaneous fat at the triceps skinfold site remaining the same in black South African adults over a five-year period. Thus, markers of adiposity increased over time, and the accumulated adipose tissue was probably distributed abdominally. Urbanicity was a predictor of change in WC for both men and women, whereas marital status was a determining factor of change in adiposity for men. We also found that increasing SES leads to elevated adiposity markers in both genders. No differences between unchanged marital status or transitioning in or out of marriage in relation to changes in adiposity markers over time were observed. However, among men, being a continuous smoker resulted in a smaller change in BMI over five years when compared to those that never smoked.

Our results, in terms of the prevalence of obesity, are comparable to rates reported in the general black South African population. For instance, the National Nutrition and Health Examination Survey described an equally high national prevalence of 39.9% obesity (BMI > 30 kg/m^2^) among black South African women [3]. The overall prevalence of obesity in this study, further confirms the rising concern that South Africa is in the nutrition-related NCD phase of the nutrition transition [3,10]. The higher prevalence of overweight subjects, obesity and abdominal obesity among women compared to the men in our study is in agreement with other investigations in sub-Saharan Africa [3,4,16,18,43]. A cultural perception among black Africans, for whom overweight or obese women are regarded as being more beautiful, symbols of happiness, well looked-after by their husbands, and being affluent, could be a reason for this continent-wide phenomenon [2,13].

The significantly greater intake of energy and fat by urban than by rural dwellers observed in our study could also be associated with the higher measures of obesity that we observed, particularly among urban women. High dietary intakes of energy and fat have been positively associated with measures of obesity [10,11,44]. Although there was no significant difference between the fat intake of men and women in our study, energy intake was greater for men. Dietary fat and energy intake were not predictors of increases in adiposity in our regression models, which could indicate that our dietary assessment method may not be sensitive enough to detect individual differences in fat and energy intake.

The level of urbanization, that is, being urban dwellers, significantly determined increases in WC of both men and women in this study. According to Cohen [45], food abundance, novelty, and variety are some of the factors that contribute to the effects of urban environments on increasing adiposity. Studies in sub-Saharan Africa have also demonstrated higher BMI in urban compared to rural people [18,43]. Abdominal and overall obesity were reported to be higher among urban Kenyans compared with their rural counterparts [6]. The picture is different in western countries, as inhabitants of rural areas have been reported to display higher measures of obesity than urban dwellers [19]. Even though measures of obesity were higher in our urban women compared to their rural counterparts, we observed a trend of increased obesity in both urban and rural areas over the five-year period (Figure 2b). This reflects findings that increase in adiposity parameters could be an indication of nutrition transition even in the rural areas with its resultant adverse effects as previously observed [10,11].

It has been reported that the association between indicators of SES and BMI varied depending on the socio-economic development of a country [5,8]. McLaren [8] used the human development index to compare low-, middle-, and high-income countries and found that the association between socio-economic indicators and obesity was mostly positive in LMICs and largely negative in rich nations. Earlier studies reported that SES was a significant predictor of BMI and that SES was inversely associated with BMI among European populations [7,46]. Our findings of a positive association between SES index and WC in both men and women are in line with studies in other LMICs [8,16]. The SES index also positively predicted increased WC in both men and women. Future studies can elaborate on our findings by gathering additional information on the ownership of household assets such as cars, televisions, and microwave ovens to create an asset index, which could complement or perhaps even increase the sensitivity of the SES index used here.

An inverse association between educational status and BMI was found among women in Sweden [7], whereas a positive association between BMI and educational status was observed in our study, as well as among Ghanaians [43]. The educational level of the women in our study was also positively associated with gain in WC. However, in our study it is important to keep in mind that the majority of the women (83%) were educated only up to primary school level. The trend was similar for the men as only 16% of them had school education beyond primary school. Our results are in keeping with another South African report; Sartorius et al. [16] observed an association between primary or secondary schooling with increased risk of obesity, whereas those with tertiary education were not at higher risk than those with no schooling.

It is well known that physical activity is inversely associated with measures of obesity [14]. Earlier studies showed that habitual physical activity levels are low in black South African women of the North West province [38,39], similar to what we found in both men and women in our study. However, urban men in the present investigation reported a higher level of physical activity than urban women. Contrary to expectations, we found that baseline physical activity levels correlated positively with BMI change in women and positively predicted BMI alterations in men. This could partly be explained by BMI being a measure of lean mass together with fat mass, and therefore, changes in BMI do not reflect increased adiposity only. Physical activity has been shown to predict lean mass accrual [47]. Moreover, physical activity did not positively correlate with nor predict any of the other adiposity markers thus corroborating our hypothesis.

Smoking is associated with central fat accumulation [15] and has been shown to have a strong negative association with the BMI of women in Europe [7] and South Africa [16]. Tobacco use had no relationship with changes in BMI or WC, but was a negative predictor of changes in triceps skinfold thickness for women in our study. For men in our study, continued smoking were associated with lower rates of increase in BMI than those that never smoked over a five-year period. In our study, changes in adiposity between those that quitted smoking, did not differ from other smoking status categories as was reported by Cois and Day [17].

In the current investigation regression models explained only between 3% and 23.1% of the variations in change in adiposity over five years. This level of explanation seems low, but is comparable to the variation of adiposity change accounted for in the WHO MONICA study [48], where only 4% of variation in WC was explained in men and 5% in women, when other anthropometric parameters were excluded. The innate restrictions of the SES index, dietary intakes and epidemiological data on physical activity may clarify the low variation explained by the regression models in our study, but also in other studies.

Being married or cohabiting (married under common law) was a significant determinant of increased adiposity (WC, triceps skinfold thickness, and BMI) of men, but not in the women. The higher percentage of obese and abdominally obese married men compared to single obese men in our study agrees with a recent report on South Africans [16]. A study in an Iranian population showed that marriage was associated with an increased risk of obesity in both genders [23]. Reports on Americans indicated that men’s and women’s weights were differently associated with marital changes [24,26]. In a cross-sectional study, married men were significantly more likely to be obese than single men, whereas marital status was not associated with obesity among women [26]. In a later report on Americans, unmarried women who married gained weight whereas single men lost more weight than married men over 10 years. The reason(s) for this phenomenon is not clear. Possible causes could be marriage-associated social environments and lifestyle changes, such as reduced physical activity, increased food consumption, and different smoking habits [23,24]. We also hypothesize that the cultural view of a larger body being the ideal among women may have influenced the men as well, and that larger married men might also be viewed as being well-cared-for by their wives. Marital transitions were also investigated in our study, because it was postulated by others that they trigger change in one’s lifestyle that might alter health behavior [24,25]. In other publications, transitions out of marriage through widowhood were more important than through divorce or transitions into marriage in triggering weight change, because the latter two are transient [25]. However, we did not observe any changes in markers of adiposity between continuous or marital transition categories in our population.

Our work has strengths and limitations. It was performed on black men and women of Tswana ancestry in one province. However, local data are important for informing weight-loss intervention that might be culture sensitive. Moreover, the results may not be generalizable to the greater South African population that also includes minority groups, which were not studied in our investigation. A strength of our study was that the sample included men and women over a wide range of BMI and that we deployed two additional indicators of adiposity (WC and triceps skinfold thickness), because BMI does not distinguish fat from muscle mass. The smaller number of male than female subjects in this study might be problematic, but low participation rates among men is common in epidemiological studies in South Africa [49]. Despite the limitations often encountered in epidemiological data, the scientific rigour of our study renders our results sound. For this reason, we believe that our findings are solid grounds upon which to make recommendations that are crucial for informing intervention and treatment efforts of excessive adiposity in the South African context.

## 5. Conclusions

Our longitudinal findings over five years showed that black African adults experienced increases in BMI and WC. Residing in an urban environment and having a higher SES played a significant role in increasing the adiposity of our participants. Marital status, among men, also seems to be a contributor to excessive adiposity. Based on the current study and other statistics on obesity in South Africa, if we do not intervene in addressing the issue, the long-term expectation is that obesity will increase further in future. We postulate that for effective evidence-informed prevention or treatment in our population, focusing on the urban living, married people, and those of higher SES—the high-risk groups identified herein—will be essential to reduce the prevalence and extent of adiposity with time. Targeted interventions might be more effective in containing the adverse consequences for health, and even death, with the associated economic burden due to excessive adiposity than general, unfocused intervention strategies.

## Figures and Tables

**Table 1:** Baseline descriptive data stratified according to sex.

Variables			Men (*n* = 365 *)	Women (*n* = 693 *)	*p* ^a^
Age at baseline (year), mean ±SD			51.9 ± 10.1	51.8 ± 10.2	0.95
Marital status % (*n*)		Living single	42.2 (152)	47.5 (317)	0.11
		Married/cohabiting	57.8 (208)	52.5 (351)	
Socio-economic variables	Stratum of urbanization % (*n*)	Urban	49.6 (181)	42.9 (297)	0.04
		Rural	50.4 (184)	57.1 (396)	
	Education % (*n*)	No formal education	41.5 (149)	38.4 (257)	0.59
		Low (1–7 years)	42.1 (151)	44.5 (298)	
		Intermediate (8–12 years)	15.3 (55)	16.6 (111)	
		High (>12 years)	1.1 (4)	0.6 (4)	
	Employed full-time		59.7 (218)	57.4 (398)	0.77
	Occupation % (*n*)	Domestic/informal worker	89.0 (325)	88.0 (610)	0.23
		Formally trained/skilled	4.1 (15)	2.6 (18)	
		Professionals	0.8 (3)	0.6 (4)	
		No answer	6.0 (22)	8.8 (61)	
	Type of roofing	Tiles, slates or reinforced concrete	3.6 (13)	3.2 (22)	0.82
		Galvanized iron	79.7 (291)	82.0 (568)	
		Asbestos	14.2 (52)	12.4 (86)	
		Scrap material	2.5 (9)	2.5 (17)	
	Electricity % (*n*)		88.5 (323)	91.3 (633)	0.23
	Piped water in house % (*n*)		45.5 (166)	36.4 (252)	0.004
	SES index score		7.83 ± 1.27	7.77 ± 1.13	0.43
Life style	Tobacco use % (*n*)		63.2 (230)	47.2 (325)	<0.001
	Physical activity score, median (interquartile range)		2.83 (2.52–3.23)	2.90 (2.57–3.25)	0.40
	Energy intake (kg), mean ±SD		8563 ± 3625	7413 ± 3512	<0.001
	Fat intake (g), mean ±SD		50.1 ± 29.5	48.0 ± 32.3	0.33
Adiposity parameters	BMI (kg/m^2^)		21.0 ± 4.32	27.6 ± 7.41	<0.001
	Height (cm)		167 ± 6.75	157 ± 6.25	<0.001
	Weight (kg)		58.7 ± 12.7	67.9 ± 18.8	<0.001
	WC (cm)		77.1 ± 10.6	82.9 ± 13.8	<0.001
	Triceps SFT (mm)		9.32 ± 6.09	22.3 ± 9.30	<0.001
	Obese: BMI >30 kg/m^2^, % (*n*)		4.1 (15)	34.9 (242)	<0.001
	Abdominal obesity *^¥^*, % (*n*)		7.4 (27)	56.1 (389)	<0.001

Normally distributed data are reported as mean ±SD, non-normally distributed data as median and interquartile range, frequencies and percentages of the group; * Sample size varies due to missing values; ^a^ Level of significance for differences between men and women; ***^¥^*** WC >80 cm for women and >94 cm for men; BMI, body mass index; SD, standard deviation; SES, socio-economic status; SFT, skinfold thickness; WC, waist circumference.

**Table 2:** Changes in adiposity over five years stratified by sex and residence.

Anthropometric Variables	Residence	Men			Women		
		Baseline	Follow-Up	Δ	Baseline	Follow-Up	Δ
Weight (kg)	Urban	58.5 ± 12.6	58.9 ± 14.1	0.46 ± 5.00	70.6 ± 19.5	71.7 ± 20.6	1.09 ± 6.36
	Rural	59.0 ± 13.0	60.1 ± 13.3	1.04 ± 4.98	65.7 ± 18.1	68.4 ± 18.5	2.52 ± 6.37
BMI (kg/m^2^)	Urban	20.9 ± 4.18	21.3 ± 4.90	0.34 ± 2.15	28.7 ± 7.58	29.4 ± 8.06	0.62 ± 2.80
	Rural	21.1 ± 4.47	21.6 ± 4.67	0.50 ± 1.77	26.7 ± 7.17	27.9 ± 7.34	1.11 ± 2.82
WC (cm)	Urban	76.6 ± 10.3	78.4 ± 11.5	1.83 ± 5.95	84.7 ± 3.45	88.2 ± 1.56	3.45 ± 7.65
	Rural	77.5 ± 10.9	77.8 ± 10.5	0.27 ± 4.13	81.4 ± 14.0	82.7 ± 13.2	1.13 ± 6.18
Triceps SFT (cm)	Urban	8.6 ± 5.50	9.2 ± 5.87	−0.6 ± 3.69	21.1 ± 8.50	23.8 ± 11.4	0.81 ± 8.17
	Rural	10.1 ± 6.81	9.4 ± 6.41	0.7 ± 4.79	23.1 ± 9.77	24.4 ± 9.98	−0.37 ± 7.98

BMI, body mass index; SFT, skinfold thickness; WC, waist circumference.

**Table 3:** Spearman correlations between changes in adiposity and baseline dietary intake, socio-economic, and lifestyle variables.

Variable		Men			Women		
		Δ BMI (kg/m^2^)	Δ WC (cm)	Δ Triceps SFT (mm)	Δ BMI (kg/m^2^)	Δ WC (cm)	Δ Triceps SFT (mm)
		(*n* = 364)	(*n* = 363)	(*n* = 358)	(*n* = 691)	(*n* = 685)	(*n* = 569)
Age (year)		0.04	0.07	0.002	−0.16 **	−0.07	−0.12 **
Socio-economic	Education level	−0.02	0.03	0.01	0.0	0.11 *	0.08
	Occupation (graded)	0.04	0.04	−0.02	0.03	0.03	−0.03
	SES index	−0.04	0.05	0.04	0.04	0.14 **	0.07
Lifestyle	Physical activity score	0.10	0.05	−0.02	0.13 *	−0.02	0.08
	Energy intake (kJ)	−0.08	0.02	0.05	−0.06	0.09 *	−0.05
	Fat intake (g)	−0.03	0.08	0.10	−0.05	0.09 *	0.02

* Significant at *p* < 0.05; ** significant at *p* < 0.01; Δ, change; BMI, body mass index; WC, waist circumference; SES, socio-economic status; SFT, skinfold thickness.

**Table 4:** Multiple regression analysis for the association between changes in adiposity variables (∆ BMI, ∆ WC, and ∆ triceps SFT in men and women, respectively) as dependent variables and predictor variables.

Predictor Variables	∆ BMI		∆ WC		∆ Triceps SFT	
	Men	Women	Men	Women	Men	Women
**Model 1: Full model with all variables**						
Baseline BMI	−0.07	−0.08	N/A	N/A	N/A	N/A
Baseline WC	N/A	N/A	−0.28 **	−0.26 **	N/A	N/A
Baseline triceps SFT	N/A	N/A	N/A	N/A	−0.48 **	−0.36 **
Baseline age	−0.08	−0.09	0.02	−0.05	−0.02	−0.06
Baseline SES index	0.003	0.05	0.13 *	0.09 *	0.10	0.10 *
Baseline physical activity score	0.11	0.05	0.02	0.02	−0.04	0.05
Baseline fat intake (g)	0.09	−0.01	−0.005	−0.04	0.06	0.06
Baseline tobacco use *0 = never used, 1 = ever used*	−0.07	−0.02	−0.09	0.01	−0.10	−0.10 *
Baseline marital status *0 = single, 1 = married/cohabiting*	0.22 **	0.03	0.27 **	0.07	0.14 *	−0.06
Stratum of urbanization *0 = rural, 1 = urban*	−0.07	0.00	0.15 *	0.23 **	0.09	0.02
Baseline menopausal status *0 = premenopausal, 1 = postmenopausal*	N/A	−0.09	N/A	−0.03	N/A	−0.10 *
Adjusted R^2^	0.054	0.024	0.131	0.100	0.228	0.129
**Model 2: Model with best fit**						
Baseline BMI	--	−0.08	N/A	N/A	N/A	N/A
Baseline WC	N/A	N/A	−0.28 **	−0.26 **	N/A	N/A
Baseline triceps SFT	N/A	N/A	N/A	N/A	−0.48 **	−0.36 **
Baseline age	−0.09	−0.09	--	−0.07	--	−0.06
Baseline SES index	--	0.05	0.12 *	0.09 *	0.12 *	0.09 *
Baseline marital status *0 = single, 1 = married/cohabiting*	0.22 **	--	0.27 **	--	0.13 *	--
Stratum of urbanization *0 = rural, 1 = urban*	--	--	0.15 *	0.22 **	0.12 *	
Baseline physical activity score	0.12 *	0.05	--	--	--	--
Baseline menopausal status *0 = premenopausal, 1 = postmenopausal*	N/A	−0.08	N/A	--	N/A	−0.11 *
Baseline tobacco use *0 = never used, 1 = ever used*	--	--	−0.09	--	--	−0.10 *
Adjusted R^2^	0.057	0.030	0.138	0.107	0.231	0.132

Numbers are beta values and data are adjusted for baseline variables: specific relevant anthropometric measures of adiposity, age, SES index, physical activity score, fat intake, energy intake, tobacco use, marital status, menopausal status (for women only) and stratum of urbanization; * *p* < 0.05; ** *p* < 0.001; ∆, change; BMI, body mass index; NA, not applicable; WC, waist circumference; SES, socio-economic status; SFT, skinfold thickness.
